# Editorial: Diabetes and cardiovascular complications: synergistic treatment approaches

**DOI:** 10.3389/fcdhc.2025.1689009

**Published:** 2025-09-24

**Authors:** Belma Pojskic

**Affiliations:** Cantonal Hospital, Medical Faculty University Zenica, Zenica, Bosnia and Herzegovina

**Keywords:** diabetes mellitus, cardiovascular risk, cardiovascular complications, integrated management, public health

The 20th century was marked by groundbreaking discoveries in organ and tissue physiology, paving the way for targeted therapies for individual diseases. In contrast, the 21st century has brought a paradigm shift in our understanding of chronic diseases, revealing intricate connections between conditions once considered distinct. Nowhere is this more evident than in the interplay between metabolic and cardiovascular systems. Where diabetes and cardiovascular disease (CVD) were once treated as separate entities, we now recognize that their shared pathophysiology demands equally integrated management strategies (1).

Recent studies featured in this Research Topic provide compelling evidence for this shift, illustrating both persistent gaps in care and innovative approaches that address the cardiometabolic continuum.

A retrospective analysis from South Africa (Mhlaba et al.) examined diabetes management in patients at high cardiovascular risk, revealing that only 28.7% achieved target HbA1c levels. Treatment challenges were particularly pronounced in certain subgroups, underscoring substantial unmet needs in current clinical practice. These findings align with contemporary guidelines advocating more ambitious treatment targets for high-risk populations (2). Beyond guideline alignment, the study calls attention to structural barriers—limited access to advanced therapies, fragmented care pathways, and inadequate patient engagement— that require strategic action to effectively translate established evidence into measurable, real-world improvements in patient outcomes.

The metabolic consequences of cancer therapies form another critical dimension of the diabetes–CVD intersection. (Avagimyan et al.) Chemotherapy-induced metabolic disturbances can exacerbate cardiovascular risk, creating a dual burden in already vulnerable patients. Emerging data suggest that certain antidiabetic agents may offer both metabolic and cardioprotective benefits in this context (3). These insights highlight the growing relevance of cardio-oncology and the need for integrated care models that anticipate and mitigate treatment-induced metabolic derangements, ultimately improving long-term survivorship and quality of life.

Genetic insights into lipid metabolism (Alieva et al.) provide valuable understanding of how inter-individual variability influences metabolic and cardiovascular outcomes. Such findings reinforce the momentum toward precision medicine approaches in diabetes care, as recognized in recent consensus statements (4). Importantly, this work moves beyond risk description—it offers a pathway to individualized therapy selection, early intervention, and more efficient allocation of resources toward those at greatest risk of adverse cardiometabolic outcomes.

A comprehensive review of incretin-based therapies (Ferhatbegović et al.) further supports their pleiotropic benefits, consolidating evidence that these agents deliver robust cardiovascular protection alongside glycemic control (5). In the context of ongoing debates about treatment sequencing in high-risk patients, these findings strengthen the rationale for prioritizing GLP-1 receptor agonists and SGLT2 inhibitors early in the therapeutic algorithm. Beyond their glucose-lowering properties, their anti-inflammatory, weight-reducing, and renal-protective effects position them as cornerstone agents in the integrated management of diabetes and CVD.

## Clinical implications and future directions

Collectively, the studies in this Research Topic support four key practice priorities:

1. Therapeutic Prioritization: Current guidelines, including the 2019 ESC Guidelines on Diabetes, Pre-Diabetes and Cardiovascular Diseases (5) and the 2022 ADA/EASD Consensus Report (6), should be strengthened to reflect the consistent benefits of SGLT2 inhibitors and GLP-1 receptor agonists across multiple high-risk populations. These landmark documents already prioritize agents with proven cardiovascular benefit, but the accumulating evidence warrants even earlier and broader implementation in clinical practice.
2. Advanced Risk Stratification: The demonstrated variability in treatment response supports development of more sophisticated risk assessment tools incorporating genetic, metabolic, and clinical parameters. As outlined in Pearson’s seminal review on precision medicine in diabetes (7), integrating these multidimensional data can guide individualized therapy selection and improve both metabolic and cardiovascular outcomes.
3. Oncological Integration: The intersection of cancer therapy and metabolic health warrants specific clinical attention and potential guideline updates regarding monitoring and prevention strategies. The 2022 ESC Cardio-Oncology Guidelines (8) provide a structured framework for surveillance, early detection, and management of cardiovascular complications in cancer patients, which could be expanded to systematically address metabolic disturbances as well.
4. Implementation Science: The persistent gaps in achieving treatment targets emphasize the need for better implementation of existing evidence, particularly in resource-limited settings. As highlighted by the ADA’s implementation framework (9), effective translation of guidelines into practice requires structured care pathways, multidisciplinary collaboration, and system-level support.

The essential implication is clear: optimal management of diabetes and CVD requires moving beyond a glucose-centric paradigm. It demands therapies with proven cardiometabolic benefits, individualized care models informed by genetic and metabolic profiling, and system-level reforms that enable effective implementation. Landmark cardiovascular outcomes trials, such as the CANVAS Program, have already demonstrated the transformative potential of targeted agents to simultaneously reduce cardiovascular events and improve metabolic control (11).

This body of work collectively advocates for a fundamental redefinition of diabetes care—from a narrow focus on glycemic control to a broader vision of metabolic intervention as cardiovascular therapy (10, 11). With the convergence of strong evidence, precision tools, and therapeutic innovations, the opportunity to improve both metabolic and cardiovascular outcomes has never been greater.
